# Circumferential resection margin as a prognostic factor after rectal cancer surgery: A large population‐based retrospective study

**DOI:** 10.1002/cam4.1662

**Published:** 2018-07-10

**Authors:** Qi Liu, Dakui Luo, Sanjun Cai, Qingguo Li, Xinxiang Li

**Affiliations:** ^1^ Department of Colorectal Surgery Fudan University Shanghai Cancer Center Shanghai China; ^2^ Department of Oncology Shanghai Medical College Fudan University Shanghai China

**Keywords:** circumferential resection margin, prognostic, rectal cancer, SEER

## Abstract

**Aim:**

This study aimed to investigate circumferential resection margin (CRM) as a prognostic factor for long‐term oncologic survival after rectal cancer surgery.

**Methods:**

Patients diagnosed with malignant rectal cancer between 1 January 2010 and 31 December 2014, from the Surveillance, Epidemiology, and End Results (SEER) program were identified for this study. The patients were divided into five CRM groups to compare the baseline characteristics and assess cancer‐specific survival (CSS): 0‐1 mm, 1.1‐2.0 mm, 2.1‐5.0 mm, 5.1‐10.0 mm, and >10 mm. The main endpoint was CSS.

**Results:**

Circumferential resection margin ≤1 mm was independently associated with 99% increased risk of cancer‐specific mortality in rectal cancer [hazard ratio (HR) = 1.990, 95% confidence interval (CI) = 1.613‐2.454, *P *<* *0.001, using CRM (1.1‐2.0 mm) as a reference]. CRM (5.1‐10.0 mm) was independently associated with 29.2% decreased risk of cancer‐specific mortality [HR = 0.708, 95% CI = 0.525‐0.954, *P *=* *0.152, using group (2.1‐5.0 mm) as reference]. CRM ≤2 mm or ≤0.4 mm was not obviously associated with CSS.

**Conclusions:**

circumferential resection margin is an independent prognostic factor in rectal cancer. Surgeons should try to maximize the CRM. Rectal cancer patients with CRM ≤1 mm should receive more postoperative attention depending on individual situation. Also, CRM should be accurately measured in millimeters in a preoperative magnetic resonance imaging or pathological report, rather than simply described as “involved” or “clear.”

## 1 INTRODUCTION

Circumferential resection margin (CRM) is the closest distance between the radial resection margin and the tumor tissue by either direct tumor spread, areas of neural or vascular invasion, or the nearest involved lymph node.^1^ Despite the routine use of preoperative chemoradiotherapy (CT) followed by total mesorectal excision (TME) for locally advanced rectal cancer, the local recurrence and mortality remain high, and the search for potential prognostic factors has become increasingly important.^2^, ^3^

While several studies showed that CRM should not be used as a prognostic factor in rectal cancer,^4^, ^5^ other studies demonstrated the importance of CRM as an independent prognostic factor of local recurrence and long‐term survival,^6^, ^7^, ^8^, ^9^ including the first report by Quirke et al^10^ suggesting that CRM might be a strong predictor of long‐term oncologic outcomes. According to the European Society for Medical Oncology (ESMO) Clinical Practice Guidelines for rectal cancer, CRM is defined as involved if it is ≤1 mm from the tumor‐free margin, leading to an increased risk of local recurrence, distant metastases, and poorer survival.^11^

Many studies considered CRM as positive when it was ≤1 mm (R1) and associated with obviously poor prognosis as compared to CRM >1 mm (R0), which was in accordance with the ESMO guidelines.^6^, ^8^, ^12^, ^13^, ^14^, ^15^, ^16^, ^17^ The criterion to define a positive CRM remains unclear. However, some researchers believed that CRM within 2 mm was associated with a negative prognosis.^18^, ^19^, ^20^, ^21^, ^22^ In addition, Kelly et al^1^ argued that a CRM clearance >5 mm should be achieved to optimize curative treatment. Recently, Beaufrère et al^23^ found that the prognosis after rectal cancer surgery was worse with a CRM ≤0.4 mm.

Given that the aforementioned studies had a relatively small sample size, the Surveillance, Epidemiology, and End Results (SEER) program conducted a large population‐based study to analyze the prognostic ability of CRM distance in rectal cancer.

## 2 PATIENTS AND METHODS

### 2.1 CRM in SEER database

The SEER database is an authoritative source of information on the most recent cancer incidence, mortality, prevalence, and lifetime risk statistics in the United States. It is a comprehensive source of population‐based information including all newly diagnosed cancer cases among people residing in SEER‐participating areas and covering approximately 28% of the US population.

The CRMs in SEER database are, expressed as the nearest tenth in millimeters (mm), the distance between the leading edge of the tumor and the nearest edge of surgically dissected margin, as recorded in the pathology report according to The American Joint Committee on Cancer (AJCC) Seventh Edition Cancer Staging Manual: the CRM is the surgically dissected nonperitonealized surface of the specimen.

### 2.2 Study design

**Figure 1:**
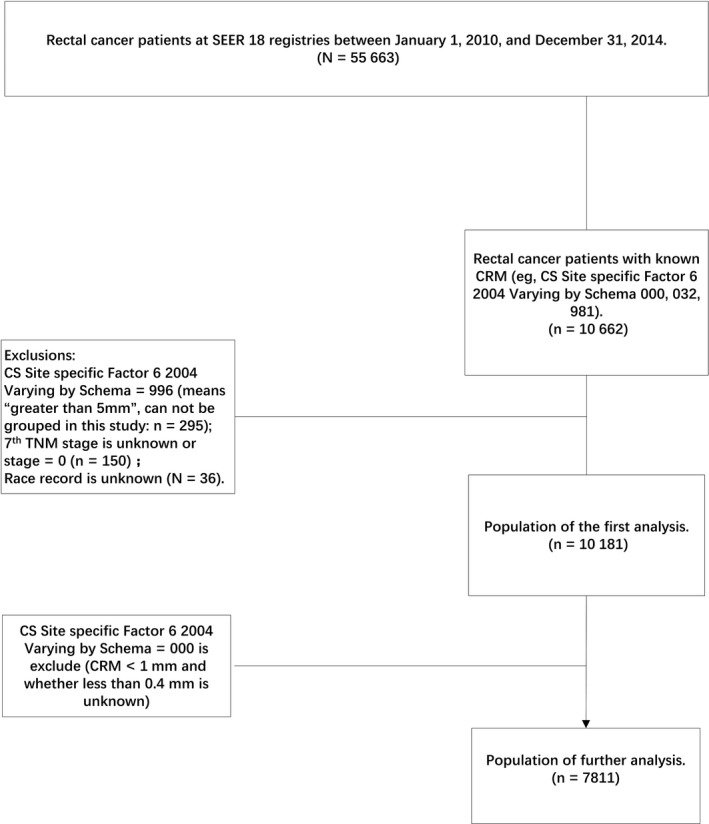
Flow diagram of patient population selection from the SEER database

Using the SEER‐Stat software (SEER*Stat 8.3.4), patients diagnosed with malignant rectal cancer between 1 January 2010 and 31 December 2014, from the SEER Program of the National Cancer Institute were identified (Figure 1). Among these patients, patients with known CRM (eg, CS Site‐Specific Factor 6, 2004, varying by Schema 000, 032, and 981) were included in the analysis. Patients with rectal cancer whose CS Site‐Specific Factor 6 was 996 (means “>5 mm”; cannot be grouped in this study), with unknown seventh AJCC stage or unknown race record, were excluded. Patients were then divided into five CRM groups: 0‐1 mm, 1.1‐2.0 mm, 2.1‐5.0 mm, 5.1‐10.0 mm, and >10 mm to compare the baseline characteristics and assess cancer‐specific survival (CSS). Next, patients whose CS Site‐Specific Factor 6 was 000 (CRM <1 mm and <0.4 mm; unknown) were also excluded. Thus, the target population for further analysis was obtained. These patients were divided into six CRM groups: ≤0.4 mm, 0.5‐1.0 mm, 1.1‐2.0 mm, 2.1‐5.0 mm, 5.1‐10.0 mm, and >10 mm.

### 2.3 Statistical analyses

Several Cox proportional hazards models were built to identify independent prognostic variables at a median survival time of 22 months (range 0‐59 months). All hazard ratios (HR) were shown with 95% confidence intervals (CI). A multivariate survival analysis was performed using a Cox proportional hazards model, including all variables associated with a *P* value <0.2 in univariate analysis. Variables including AJCC stage, tumor size, age at diagnosis, race, gender, year of diagnosis, and grade were included in the Cox multivariate survival analysis. The TNM staging used in the present study was the seventh edition of the AJCC cancer staging system, the newest TNM staging that could be obtained from the SEER database. The primary outcome of interest was CSS. The Kaplan‐Meier survival curves were used to evaluate the prognostic prediction of different factors. The log‐rank tests were used to assess statistical significance. All tests were two sided, and *P* values <0.05 were considered statistically significant. Statistical analysis was performed using Statistic Package for Social Science (SPSS) version 22 (SPSS Inc., IL, USA).

## 3 RESULTS

### 3.1 Patient characteristics of the overall cohort

A total of 10 181 patients with rectal cancer after surgery were identified from the SEER database. The baseline demographic characteristics of the patients are summarized in Table 1. A total of 4232 (41.6%) patients whose CRM was between 0 and 1 mm were included in the analyses. The overall cohort showed that higher AJCC stages (*P *<* *0.001), larger tumor size (*P *<* *0.001), black people (*P *<* *0.001), earlier year of diagnosis (*P *<* *0.001), and higher grades (*P *<* *0.001) were associated with a CRM between 0 and 1 mm. Differences in other characteristics were not significant.

**Table 1:** Comparison of baseline characteristics by various circumferential resection margins

Variable	Distance to circumferential resection margin					*P* value
	0‐1.0 mm	1.1‐2.0 mm	2.1‐5.0 mm	5.1‐10.0 mm	>10.0 mm	
AJCC stage						
Stage I	620 (32.9%)	214 (11.3%)	355 (18.8%)	166 (8.8%)	531 (28.2%)	<0.001
Stage II	1071 (39.3%)	320 (11.8%)	428 (15.7%)	274 (10.1%)	630 (23.1%)	
Stage III	1772 (41.1%)	482 (11.2%)	671 (15.6%)	426 (9.9%)	957 (22.2%)	
Stage IV	769 (60.8%)	110 (8.7%)	127 (10.0%)	84 (6.6%)	174 (13.8%)	
Tumor size						
≤5 cm	2379 (37.9%)	704 (11.2%)	1039 (16.6%)	602 (9.6%)	1549 (24.7%)	<0.001
>5 cm	1499 (47.7%)	315 (10.0%)	444 (14.1%)	283 (9.0%)	601 (19.1%)	
Unknown	354 (46.2%)	107 (14.0%)	88 (12.8%)	65 (8.5%)	142 (18.5%)	
Age at diagnosis (y)						
≤60	1903 (41.4%)	504 (11.0%)	700 (15.2%)	433 (9.4%)	1061 (23.1%)	0.757
>60	2329 (41.7%)	622 (11.1%)	881 (15.8%)	517 (9.3%)	1231 (22.1%)	
Race						
White	3388 (40.9%)	932 (11.2%)	1313 (15.8%)	779 (9.4%)	1876 (22.6%)	<0.001
Black	408 (51.3%)	84 (10.6%)	104 (13.1%)	54 (6.8%)	145 (18.2%)	
Other	436 (39.7%)	110 (10.0%)	164 (14.9%)	117 (10.7%)	271 (24.7%)	
Gender						
Male	2543 (42.0%)	688 (11.4%)	907 (15.0%)	562 (9.3%)	1354 (22.4%)	0.287
Female	1689 (40.9%)	438 (10.6%)	674 (16.3%)	388 (9.4%)	938 (22.7%)	
Year of diagnosis						
2010	895 (48.5%)	225 (12.2%)	290 (15.7%)	157 (8.5%)	280 (15.2%)	<0.001
2011	874 (42.9%)	229 (11.3%)	317 (15.6%)	172 (8.5%)	443 (21.8%)	
2012	873 (41.3%)	216 (10.2%)	325 (15.4%)	208 (9.8%)	491 (23.2%)	
2013	803 (38.8%)	220 (10.6%)	317 (15.3%)	213 (10.3%)	516 (24.9%)	
2014	787 (37.2%)	236 (11.1%)	332 (15.7%)	200 (9.4%)	562 (26.5%)	
Grade						
Grade I	279 (43.7%)	75 (11.8%)	110 (17.2%)	61 (9.6%)	113 (17.7%)	<0.001
Grade II	2835 (38.1%)	859 (11.5%)	1217 (16.3%)	730 (9.8%)	1806 (24.3%)	
Grade III	725 (55.6%)	116 (8.9%)	165 (12.7%)	88 (6.7%)	210 (16.1%)	
Grade IV	170 (60.1%)	25 (8.8%)	27 (9.5%)	18 (6.4%)	43 (15.2%)	
Unknown	223 (43.8%)	51 (10.0%)	62 (12.2%)	53 (10.4%)	120 (23.6%)	

### 3.2 R1 CRM was strongly associated with poor survival in rectal cancer

The median follow‐up duration for the overall cohort was 22 months (range 0‐59 months). At the end of the follow‐up, 1262 (12.4%) patients died of rectal cancer.

A multivariate analysis was conducted to identify the variables independently associated with CSS in the overall cohort. The results of multivariate analyses by Cox regression are detailed in Table 2. R1 CRM was found to be independently associated with CSS of 10 181 patients with rectal cancer and had a 99.0% increased risk of cancer‐specific mortality [HR = 1.990, 95% CI = 1.613‐2.454, *P *<* *0.001, using group (1.1‐2.0 mm) as a reference]. In addition, Table 2 shows that lower AJCC stages, younger age, and lower grades were independent protective factors.

**Table 2:** Multivariate Cox regression analyses of CSS to study CRM ≤1 mm

Variable	Reference	Characteristic	Cancer‐specific survival		
			HR (95%CI)	SE	*P* value
CRM	1.1‐2.0 mm	0‐1.0 mm	1.990 (1.613‐2.454)	0.107	<0.001
		2.1‐5.0 mm	1.106 (0.855‐1.429)	0.131	0.443
		5.1‐10.0 mm	0.783 (0.569‐1.075)	0.162	0.131
		>10.0 mm	0.825 (0.637‐1.070)	0.132	0.147
AJCC stage	Stage I	Stage II	2.426 (1.769‐3.327)	0.161	<0.001
		Stage III	4.759 (3.538‐6.400)	0.151	<0.001
		Stage IV	15.909 (11.773‐21.499)	0.154	<0.001
Tumor size	Unknown	≤5 cm	0.803 (0.645‐1.000)	0.112	0.050
		>5 cm	1.129 (0.904‐1.411)	0.114	0.284
Age at diagnosis (y)	≤60	>60	1.777 (1.584‐1.993)	0.058	<0.001
Year of diagnosis	2010	2011	1.124 (0.973‐1.299)	0.074	0.111
		2012	0.884 (0.747‐1.046)	0.086	0.152
		2013	1.014 (0.832‐1.237)	0.101	0.887
		2014	0.830 (0.611‐1.128)	0.156	0.234
Grade	Grade I	Grade II	1.286 (0.942‐1.757)	0.159	0.113
		Grade III	2.195 (1.584‐3.041)	0.166	<0.001
		Grade IV	2.793 (1.914‐4.074)	0.193	<0.001
		Unknown	1.439 (0.963‐2.150)	0.205	0.076

**Figure 2:**
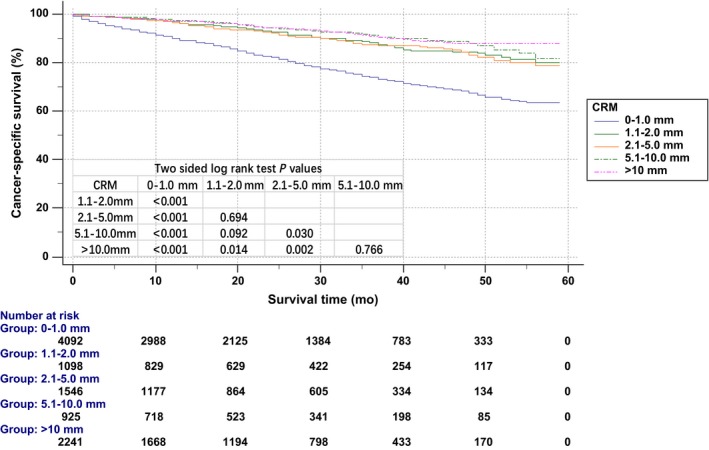
Kaplan‐Meier cancer‐specific survival curve according to circumferential resection margin (CRM)

Kaplan‐Meier CSS curves were used to analyze the prognosis of different CRMs (Figure 2). Group (0‐1.0 mm) was associated with poorer CSS (90.0% for 1‐year CSS and 73.8% for 3‐year CSS). The 3‐year CSS of group (1.1‐2.0 mm), group (2.1‐5.0 mm), group (5.1‐10.0 mm), and group (>10.0 mm) was 88.2%, 87.3%, 91.4%, and 90.8%, respectively. However, the differences between group (1.1‐2.0 mm) and group (2.1‐5.0 mm), group (1.1‐2.0 mm) and group (5.1‐10.0 mm), and group (5.1‐10.0 mm) and group (>10.0 mm) were not statistically significant. The Cox multivariate CSS analysis also showed no significant difference between group (2.1‐5.0 mm) and group (1.1‐2.0 mm) [HR = 0.905, 95% CI = 0.700‐1.169, *P *=* *0.443, using group (2.1‐5.0 mm) as reference] (Table S1). However, group (5.1‐10.0 mm) had more favorable prognosis as compared to group (2.1‐5.0 mm) [HR = 0.708, 95% CI = 0.525‐0.954, *P *=* *0.152, using group (2.1‐5.0 mm) as reference].

**Figure 3:**
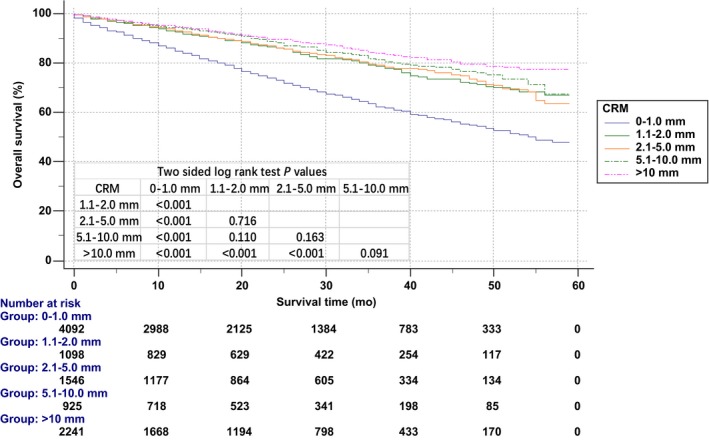
Kaplan‐Meier overall survival curve according to circumferential resection margin (CRM)

Kaplan‐Meier OS curves were also used to analyze the prognosis of different CRMs (Figure 3). Group (0‐1.0 mm) was associated with poorer OS (84.9% for 1‐year OS and 62.4% for 3‐year OS). The 3‐year OS of group (1.1‐2.0 mm), group (2.1‐5.0 mm), group (5.1‐10.0 mm), and group (>10.0 mm) was 78.5%, 79.0%, 81.3%, and 83.8%, respectively. However, the differences between group (1.1‐2.0 mm) and group (2.1‐5.0 mm), group (1.1‐2.0 mm) and group (5.1‐10.0 mm), group (2.1‐5.0 mm) and group (5.1‐10.0 mm), and group (5.1‐10.0 mm) and group (>10.0 mm) were not statistically significant.

### 3.3 Further analysis of R1 CRM

Patients whose CRM was not known to be <0.4 mm were excluded. Hence, 7811 patients with rectal cancer were identified for further analysis of R1 CRM. R1 CRM was further divided into group (≤0.4 mm) and group (0.5‐1.0 mm).

A multivariate analysis was conducted to identify whether the CSS was different between group (≤0.4 mm) and group (0.5‐1.0 mm) in this target population. The results of multivariate analyses by Cox regression are detailed in Table 3. The difference between group (≤0.4 mm, n = 741) and group (0.5‐1.0 mm, n = 576) was HR = 0.834, 95% CI 0.645‐1.078, using group (≤0.4 mm) as reference, but it was not statistically significant (log‐rank test, *P *=* *0.166).

**Table 3:** Multivariate Cox regression analyses of CSS to study CRM ≤0.4 mm

Variable	Reference	Characteristic	Cancer‐specific survival		
			HR (95%CI)	SE	*P* value
CRM	≤0.4 mm	0.5‐1.0 mm	0.834 (0.645‐1.078)	0.131	0.166
		1.1‐2.0 mm	0.704 (0.536‐0.924)	0.139	0.012
		2.1‐5.0 mm	0.773 (0.602‐0.992)	0.127	0.043
		5.1‐10.0 mm	0.543 (0.397‐0.741)	0.159	<0.001
		>10.0 mm	0.577 (0.448‐0.742)	0.129	<0.001
AJCC stage	Stage I	Stage II	2.041 (1.388‐3.001)	0.197	<0.001
		Stage III	4.361 (3.060‐6.216)	0.181	<0.001
		Stage IV	15.773 (10.949‐22.722)	0.186	<0.001
Tumor size	Unknown	≤5 cm	0.941 (0.685‐1.292)	0.162	0.707
		>5 cm	1.283 (0.927‐1.775)	0.166	0.133
Age at diagnosis (y)	≤60	>60	1.962 (1.674‐2.298)	0.081	<0.001
Grade	Grade I	Grade II	1.125 (0.738‐1.714)	0.215	0.583
		Grade III	2.077 (1.331‐3.241)	0.227	0.001
		Grade IV	2.899 (1.685‐4.987)	0.277	<0.001
		Unknown	1.027 (0.584‐1.805)	0.288	0.926

## 4 DISCUSSION

While CRM is widely accepted as a strong independent prognostic factor of long‐term oncologic survival, the criterion used to define a positive CRM remains controversial.^16^ Therefore, this retrospective study was conducted to assess the influence of CRM on prognosis after rectal cancer surgery. The present study included more than 10 000 patients with rectal cancer, which greatly exceeded the number of cases in previous studies and hence the results of this study are persuasive and depict real‐world scenario.

R1 CRM was found to be an independent factor for poor prognosis and had 99.0% increased risk of cancer‐specific mortality as compared to group (1.1‐2.0 mm). This is consistent with most studies on the prognostic prediction of CRM.^6^, ^7^, ^8^, ^12^, ^13^, ^14^, ^15^, ^16^

However, some researchers argued that CRM <2 mm was associated with a negative prognosis.^18^, ^19^, ^20^, ^21^, ^22^ A CRM clearance of >5 mm and 0.4 mm was proposed by Kelly et al^1^ in 2009 and Beaufrère et al^23^ in 2017, respectively. Relevant analyses were also conducted in this study to assess previous study results. The Cox multivariate analysis showed that the differences in CSS between group (1.1‐2.0 mm) and group (2.1‐5.0 mm) were not statistically significant. Yet, group (5.1‐10.0 mm) had 29.2% decreased cancer‐specific mortality as compared to group (2.1‐5.0 mm). After excluding the patients whose CRM was <0.4 mm or unknown, the Cox multivariate analysis showed no statistically significant difference between group (≤0.4 mm) and group (0.5‐1 mm). Given the large sample size in the present study, it was believed that 2 and 0.4 mm were not optimal cutoff values, in partial agreement with Kelly et al. In the study by Kelly et al, the multivariate analysis showed 32.4% increased cancer‐specific mortality in group (>1 and ≤5 mm) as compared to group (>5 and ≤10 mm), which was similar to the result of the present study. However, there was no obvious difference in CSS between group (0‐1.0 mm) and group (1.1‐2.0 mm) in the present study.

The treatment modalities have dramatically changed in the recent years. The introduction of newer surgical techniques (TME and laparoscopy) and neoadjuvant chemoradiotherapy have reduced the incidence of positive CRMs.^9^ Well‐performed TMEs with a resection margin on the mesorectal plane showed <10% of margin positivity.^24^, ^25^ The European Organization for Research and Treatment of Cancer trial showed that neoadjuvant radiochemotherapy had 9% decreased margin positivity as compared to short‐course radiotherapy.^26^

MRI is the most accurate method for preoperative diagnosis of rectal cancer and can detect tumor invasion.^27^ The results of the present study suggested that neoadjuvant radiochemotherapy should be considered if the distance of tumor and the mesorectal fascia is predicted to be <1 mm by preoperative MRI in rectal cancer. While some recent studies have reported that postoperative treatment did not improve outcomes in this situation,^28^, ^29^, ^30^ we hypothesize that CRM could guide postoperative treatment in combination with preoperative MRI assessment and neoadjuvant chemotherapy.^31^, ^32^, ^33^ Also, the prognosis is typically better when the distance of the tumor is larger from the radial resection margin. Therefore, surgeons should try to maximize the CRM and at least 1 mm of CRM should be reached. Given the 32.4% increased cancer‐specific mortality in group (>1, ≤5 mm) as compared to group (>5, ≤10 mm), 5 mm of CRM should also be considered. Whether patients with the distance between tumor and the mesorectal fascia predicted as less than 5 mm by preoperative MRI need neoadjuvant chemoradiotherapy or with CRM ≤5 mm need more intensive postoperative attention should depend on the individual situations.

The present study also found that higher AJCC stages, larger tumor size, black people, earlier year of diagnosis, and higher grades were associated with a CRM between 0 and 1 mm, resulting in poor prognosis. AJCC stage, tumor size, and tumor grade are known prognostic factors in rectal cancer, adding to the evidence that CRM is strongly associated with the prognosis in rectal cancer.^34^, ^35^ The incidence of R1 CRM is reducing every year due to the improvements in treatment. Black people are more likely to achieve R1 CRM and should receive adequate attention. This is attributed to the financial conditions and biological differences between races.

This study was the largest till date and included more than 10 000 patients for the analyses of the prognostic prediction of CRM and was the first to simultaneously analyze postoperative R1 CRM and R0 CRM in depth.

This study had several limitations. First, the SEER database lacked the data on local recurrence, which is an important factor that influences the survival of rectal cancer. However, patients with an R1 CRM often die from metastatic disease before local recurrence.^7^, ^36^ In addition, definitions of local recurrence were different in previous studies, making it difficult to examine the prognosis of different CRMs. Therefore, CSS is thought to be a more robust endpoint to assess the prognostic prediction of CRM.^37^ Second, the lack of factors influencing the treatment might have affected the results to some extent. However, the large sample size could offset this influence. Further, the lack of preoperative treatment had minimal effect since CRM in the present study was measured post‐operation and would have improved after preoperative treatment. The longest follow‐up duration was only 59 months, not exceeding 5 years. Besides, the present analysis was solely based on retrospective data. Hence, prospective clinical studies on CRM are needed.

In summary, CRM is an independent prognostic factor in rectal cancer, and surgeons should try to maximize the CRM. R1 CRM indicates a poor prognosis. Patients with rectal cancer having R1 CRM should receive more postoperative attention. Also, 5 mm of CRM should be adequately monitored and further investigated. The closest distance between the radial resection margin and the tumor tissue should be accurately measured in millimeters in preoperative MRI or pathological report, rather than simply described as “involved” or “clear.” This may provide better treatment guidelines for clinicians.

## CONFLICT OF INTEREST

The authors declare no potential conflicts of interest.
