# Supplementary material for: Circumferential resection margin as a prognostic factor after rectal cancer surgery: A large population‐based retrospective study
Source: Cancer Med. 2018 Jul 10;7(8):3673–81. doi: 10.1002/cam4.1662 (PMC6089167; doi:10.1002/cam4.1662)
Supplement: Supplementary file 1 [file CAM4-7-3673-s001.docx]

**Supplementary Table. Multivariate Cox regression analyses of CSS to study CRM ≤2 mm and CRM ≤5 mm.**

| **Variable** | \| **Reference** \| \| --- \| \| | \| **Characteristic** \| \| --- \| \| | **Cancer-specific survival** | | |
| --- | --- | --- | --- | --- | --- | --- | --- |
|  |  |  | **HR(95%CI)** | **SE** | **P value** |
| **CRM** | 2.1-5.0mm | 0-1.0mm | 1.800(1.504-2.153) | 0.092 | <0.001 |
|  |  | 1.1-2.0mm | 0.905(0.700-1.169) | 0.131 | 0.443 |
|  |  | 5.1-10.0mm | 0.708(0.525-0.954) | 0.152 | 0.023 |
|  |  | >10.0mm | 0.747(0.590-0.944) | 0.120 | 0.015 |
| **AJCC stage** | Stage Ⅰ | Stage Ⅱ | 2.426(1.769-3.327) | 0.161 | <0.001 |
|  |  | Stage Ⅲ | 4.759(3.538-6.400) | 0.151 | <0.001 |
|  |  | Stage Ⅳ | 15.909(11.773-21.499) | 0.154 | <0.001 |
| **Tumor size** | Unknown | ≤5cm | 0.803(0.645-1.000) | 0.112 | 0.050 |
|  |  | >5cm | 1.129(0.904-1.411) | 0.114 | 0.284 |
| **Age at diagnosis (years)** | ≤60 | >60 | 1.777(1.584-1.993) | 0.058 | <0.001 |
| **Year of diagnosis** | 2010 | 2011 | 1.124(0.973-1.299) | 0.074 | 0.111 |
|  |  | 2012 | 0.884(0.747-1.046) | 0.086 | 0.152 |
|  |  | 2013 | 1.014(0.832-1.237) | 0.101 | 0.887 |
|  |  | 2014 | 0.830(0.611-1.128) | 0.156 | 0.234 |
| **Grade** | Grade Ⅰ | Grade Ⅱ | 1.286(0.942-1.757) | 0.159 | 0.113 |
|  |  | Grade Ⅲ | 2.195(1.584-3.041) | 0.166 | <0.001 |
|  |  | Grade Ⅳ | 2.793(1.914-4.074) | 0.193 | <0.001 |
|  |  | Unknown | 1.439(0.963-2.150) | 0.205 | 0.076 |
